# A retrospective review of the management and outcomes of patients diagnosed with complex regional pain syndrome type II using electrodiagnostic findings

**DOI:** 10.1080/24740527.2023.2242892

**Published:** 2023-08-01

**Authors:** Fraser Alexander MacRae, Eve Boissonnault, Paul Winston

**Affiliations:** a Health Sciences, Western University, London, Canada; b Vancouver Island Health Authority, Victoria, Canada; c Physical Medicine and Rehabilitation, University of Montreal, Montreal, Canada; d Faculty of Medicine, University of British Columbia, Victoria, Canada

**Keywords:** CRPS, neuropathy, carpal tunnel syndrome, electrodiagnosis

## Abstract

**Objectives:**

The objective of this study was to assess the outcomes of the use of electrodiagnosis in the diagnosis and management of discrete nerve injuries in patients with complex regional pain syndrome (CRPS).

**Design:**

This study is a secondary retrospective cohort analysis of patients diagnosed with CRPS from a single outpatient physical medicine and rehabilitation clinic and included all patients who had abnormal electrodiagnostic findings, in addition to CRPS.

**Results:**

Sixty patients of 248 diagnosed with CRPS underwent electrodiagnosis, 41 of whom had abnormal electrodiagnostic findings indicating a discrete nerve injury. Only 51% of the 41 referrals had indicated the suspicion of a nerve injury. Nearly all patients had undergone physiotherapy. Forty-one percent responded to treatment with oral prednisone alone, 54% had a functional improvement after a combination of treatments including corticosteroids, and 5% improved with treatments that did not involve corticosteroids. Surgical interventions for nerve injuries were required for 34% of patients in the cohort. All surgeries involved the median or ulnar nerve, with the exception of one fibular nerve. After treatment, 39 of 41 patients had functional recoveries or better.

**Conclusions:**

Electrodiagnosis can inform diagnosis of nerve injury and direct intervention including the need for surgical intervention. Electrodiagnosis should be considered for patients with initial signs of concomitant discrete nerve injury or with CRPS who are not responding to treatments because a nerve injury may be underlying.

**What is Known**

Complex Regional Pain Syndrome (CRPS) is a poorly understood pain condition. CRPS has been divided into two subtypes, the second subtype involves a discrete nerve injury with pain that extends beyond the territory of the nerve injury.

**What is New**

We observed that nerve injuries that may require surgical intervention are diagnosed just over half of the time upon initial assessment in patients with suspected CRPS. We observed that nerve injuries frequently required specifically directed interventions in place of or in conjunction with CRPS treatments. We suggest that electrodiagnosis is an important part of the triage protocol for CRPS II to reveal discrete nerve injuries that may be hidden. We recommend that electrodiagnosis be considered for patients with initial signs of concomitant discrete nerve injury or for CRPS patients who do not improve with medical therapies.

## Plain Language Summary

CRPS typically occurs after an injury or inciting event and can be accompanied by severe pain, sensitivity, swelling, changes in the color of the skin, changes in the texture of the skin, changes in hair and nail growth, weakness, muscle wasting, range of motion reduction, and temperature changes in the affected area. Little is known about type II CRPS, which may present with all of the same signs and symptoms as above but with damage to a nerve in the area. This study examined cases of CRPS where patients had electrodiagnostic findings that suggested a nerve injury. We found that the referring physicians reported nerve injuries in just over half of the cases. Oral prednisone was often the chosen treatment for these patients, although many required additional treatments, including surgery, to address their nerve injury. We recommend that electrodiagnosis be considered for patients with CRPS who are not responding to corticosteroids because a nerve injury could be preventing CRPS symptoms from resolving. When the CRPS trigger was a trauma that likely simultaneously injured the nerve, patients needed to have surgery more frequently than those with atraumatic onsets.

## RÉSUMÉ

**Objectifs:** L’objectif de cette étude était d’évaluer les résultats de l’utilisation de l’électrodiagnostic pour le diagnostic et la prise en charge des lésions nerveuses discrètes chez les patients souffrant du syndrome de la douleur régionale complexe (SDRC).

**Devis:** Cette étude est une analyse de cohorte rétrospective secondaire de patients ayant reçu un diagnostic de SDRC d’une seule clinique de médecine physique et de réadaptation ambulatoire et incluait tous les patients pour lesquels les résultats de l’électrodiagnostic étaient anormaux, en plus du SDRC.

**Résultats:** Soixante patients sur les 248 ayant reçu un diagnostic de SDRC ont subi un électrodiagnostic, parmi lesquels 41 ont obtenu des résultats anormaux indiquant une lésion nerveuse discrète. Il était indiqué qu’une lésion nerveuse était soupçonnée pour seulement 51 % des 41 références. Presque tous les patients avaient reçu des traitements de physiothérapie. Tandis que 41 % ont répondu au traitement par prednisone orale seule, 54 % ont présenté une amélioration fonctionnelle après une combinaison de traitements comprenant des corticostéroïdes, et 5 % ont présenté une amélioration avec des traitements qui n’impliquaient pas de corticostéroïdes. Des interventions chirurgicales pour les lésions nerveuses ont été nécessaires pour 34 % des patients de la cohorte. Toutes les chirurgies concernaient le nerf médian ou ulnaire, à l’exception d’un nerf fibulaire. Après le traitement, 39 des 41 patients ont connu des récupérations fonctionnelles ou mieux.

**Conclusions:** L’électrodiagnostic peut éclairer le diagnostic de lésion nerveuse et l’intervention directe, y compris la nécessité d’une intervention chirurgicale. L’électrodiagnostic doit être envisagé pour les patients présentant des signes initiaux de lésions nerveuses discrètes concomitantes ou qui sont atteints du SDRC mais ne répondent pas aux traitements parce qu’une lésion nerveuse peut être sous-jacente.

Ce qui est connu

Le syndrome douloureux régional complexe (SDRC) est une affection douloureuse mal comprise. Le SDRC a été divisé en deux sous-types, le deuxième sous-type implique une lésion nerveuse discrète avec une douleur qui s’étend au-delà du territoire de la lésion nerveuse.

Ce qui est nouveau

Nous avons observé que les lésions nerveuses pouvant nécessiter une intervention chirurgicale sont diagnostiquées un peu plus de la moitié du temps lors de l’évaluation initiale des patients chez lesquels on soupçonne un SDRC. Nous avons observé que les lésions nerveuses nécessitaient souvent des interventions spécifiquement dirigées à la place des traitements pour le SDRC ou en conjonction avec ceux-ci. Nous avançons que l’électrodiagnostic constitue une partie importante du protocole de triage pour le SDRC-II afin de révéler des lésions nerveuses discrètes qui peuvent être cachées. Nous recommandons que l’électrodiagnostic soit envisagé pour les patients présentant des signes initiaux de lésion nerveuse discrète concomitante ou pour les patients atteints du SDRC dont l’état ne s’améliore pas avec les traitements médicaux.

## Résumé vulgarisé

Le SDRC survient généralement après une blessure ou un événement déclencheur et peut s’accompagner d’une douleur intense, de sensibilité, d’enflure, de changements dans la couleur et la texture de la peau, de changements dans la croissance des cheveux et des ongles, de faiblesse, d’une atrophie musculaire, d’une réduction de l’amplitude des mouvements et d’un changement de la température dans la région touchée. On sait peu de choses sur le SDRC de type II, qui peut se présenter avec tous les mêmes signes et symptômes que ci-dessus, mais avec des lésions nerveuses dans la région.

Cette étude a examiné des cas de SDRC où les résultats de l’électrodiagnostic des patients laissaient croire à une lésion nerveuse mineure. Nous avons constaté que les médecins référents signalaient des lésions nerveuses dans un peu plus de la moitié des cas. La prednisone orale était souvent le traitement choisi pour ces patients, bien qu’un grand nombre d’entre eux aient dû se soumettre à des traitements supplémentaires, y compris la chirurgie, pour traiter leur lésion nerveuse. Nous recommandons que l’électrodiagnostic soit envisagé pour les patients atteints de SDRC qui ne répondent pas aux corticostéroïdes parce qu’une lésion nerveuse pourrait empêcher les symptômes du SDRC de se résorber. Lorsque l’élément déclencheur du SDRC était un traumatisme ayant vraisemblablement endommagé le nerf en même temps, les patients ont dû subir une intervention chirurgicale plus fréquemment que ceux pour qui le déclenchement était d’origine atraumatique.

## Introduction

Complex regional pain syndrome (CRPS) remains a debated and poorly understood pain condition. CRPS may follow various inciting injuries, most commonly fractures of the distal upper extremity.^1–3^ CRPS presents as a regional pain that is out of proportion to the inciting event, accompanied by allodynia, dysesthesia, temperature asymmetry, edema, and often decreased function.^4,5^ The International Association Study for the Pain (IASP) criteria (also known as the Budapest criteria) remain the most commonly used assessment and divides CRPS into two types: type I and type II. CRPS II is less common and presents similarly to CRPS I but is associated with discrete nerve damage.

**Figure 1.**
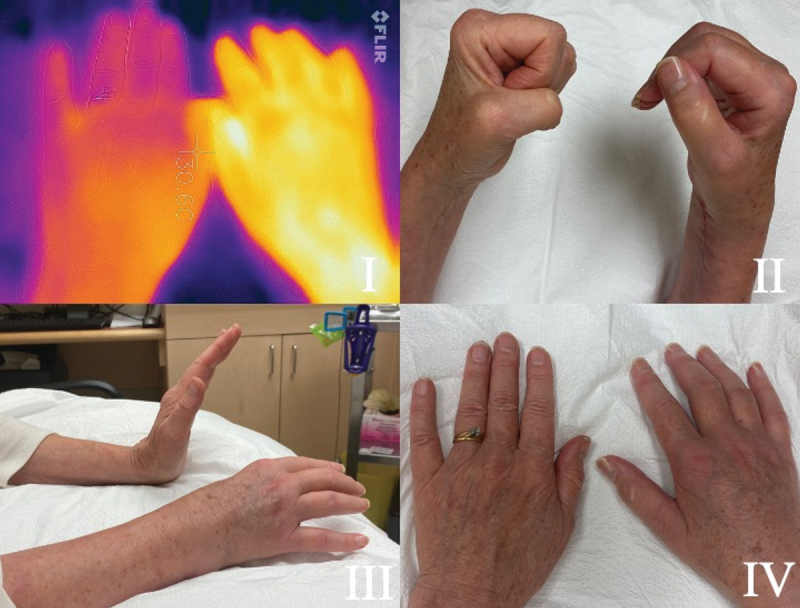
(a) A 64-year-old female presented two months after ORIF for a distal right radial and ulnar fracture. There is involvement of the wrist and all fingers. I. FLIR ONE imagery of the dorsal aspect of each hand shows temperature asymmetry. The affected side is notably warmer than the unaffected. A II,III, IV show the reduced range of motion and swelling of the joints. (b) After three weeks on prednisone. The temperature on FLIR imaging (1BI) , swelling and range of motion (1B II III) have improved. The wasting of the first dorsal interossei (FDI) (1B IV) and weak intrinsic muscles is now apparent. Nerve conductions reveal the ulnar nerve has a small amplitude of 3mV, with no slowing at the elbow. The ulnar sensory was normal. The EMG to the FDI showed florid denervation, with no motor units recruiting. The adductor digiti minimi had 2+ denervation with reduced polyphasic recruitment, while the flexor carpi ulnaris was normal. Ulnar nerve entrapment at the wrist was diagnosed. Ulnar nerve decompression was performed at the wrist. Full resolution of all symptoms was achieved.

CRPS II may not present as a territorial pain typical of neurological injuries. In the presence of overt trauma, such as a fracture, and its sequelae, physicians may not recognize a discrete neurologic injury and may not investigate for one. The regional involvement of CRPS that spreads beyond an expected joint or dermatome further adds to the diagnostic challenges, potentially hiding the presence of a discrete nerve injury (Figure 1).^4^ Clinicians must perform a neurological examination, electrodiagnostic studies, or another objective test to diagnose CRPS II.^4^ Typically, practitioners forgo electrodiagnostic studies unless paresthesia, numbness, or autonomic dysfunction are present in addition to the CRPS signs and symptoms. Patient intolerance early on usually prevents the assessment. The early onset of swelling and edema, common in CRPS cases, may hide the tell-tale sign of atrophy in the distal limbs. Ideally, CRPS should be treated within months of onset to modify the disease process. Corticosteroids, such as oral prednisone, have been recommended and endorsed as first-line treatment for subacute CRPS because of their anti-inflammatory properties. Harden et al. opined that oral corticosteroids are the only anti-inflammatory drugs for which there is direct clinical trial evidence in CRPS (level 1 evidence).^6^ Two recent review articles have supported their effectiveness in reducing CRPS symptoms.^7,8^

Electrodiagnosis is not required for the Budapest criteria. This study evaluated the use of electrodiagnosis in a cohort of patients diagnosed with CRPS. The electrodiagnosis was used at the discretion of the treating physical medicine and rehabilitation physician to identify the presence of discrete nerve injuries when a nerve injury was suspected, to direct management of the CRPS. Nerve-specific treatments included corticosteroids for both CRPS type I and the neuritis of CRPS II as well as for surgical intervention.

## Methods

The local research ethics board (Vancouver Island Health Authority; harmonized review with the University of Victoria and University of British Columbia) approved this retrospective chart review study (Protocol H21-01520) and issued a consent waiver.

### Study Methodology

**Figure 2.**
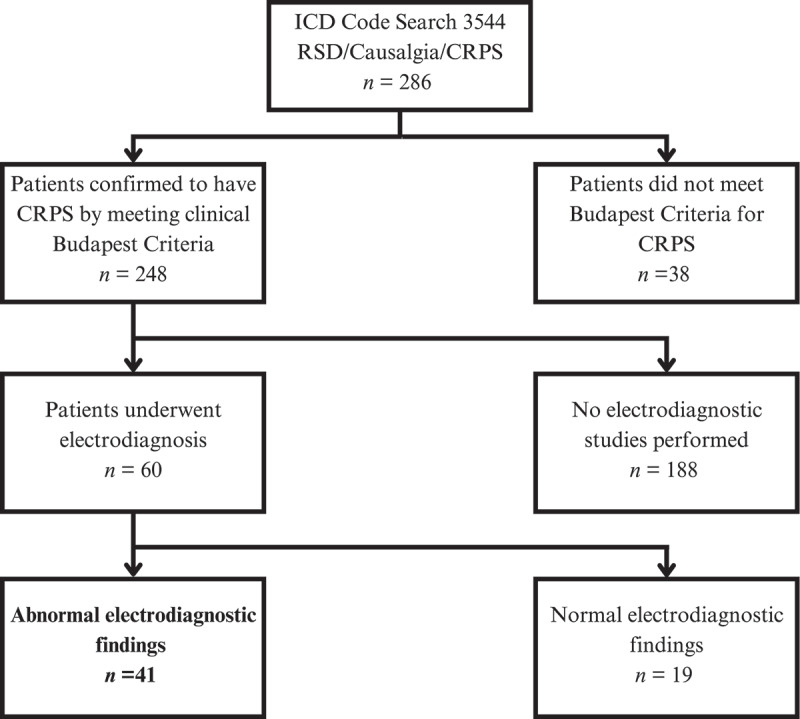
The screening process and participant selection.

This study conforms to all Strengthening the Reporting of Observational Studies in Epidemiology guidelines and reports the required information accordingly.^9^ An *International Classification of Diseases* (ICD) code search of a single physiatrist’s electronic medical records identified any charts flagged for CRPS (causalgia) between May 2013 and September 2021. A second search identified any charts that included electrodiagnostic studies. Figure 2 describes the participant selection process. Study data were collected and managed using Research Electronic Data Capture (REDCap).^10^

All charts identified by the ICD code searches were read in their entirety to determine whether they met the inclusion criteria. The results of the electrodiagnostic studies in all charts that met the inclusion criteria were reviewed and findings were recorded by the study team. Data collected for the study included demographic information, date of injury and consultation, findings of nerve conduction studies (including electrodiagnosis), treatment(s), and outcome. Outcomes were assessed using the scale provided in Table 1.

**Table 1.** Criteria for assessing patient outcomes.

Complete resolution: the patient had no residual symptoms. These patients no longer met the IASP criteria for CRPS. Near-complete resolution: the patient had very few residual symptoms that were relatively minor, such as joint stiffness. These patients met some components of the IASP criteria for CRPS. Patients in this category had a functional recovery, meaning that they are now capable of performing activities of daily living, not limited by pain or lack of function. These patients no longer require medical treatment. Some of these patients still met the Valencia criteria for CRPS with remission of some features; others may have had residual symptoms of a traumatic inciting event prevent a full recovery. Partial resolution: the patient had several residual symptoms. All or some leftover symptoms remain problematic for the patients in this category. All of these patients had a secondary treatment prescribed. These patients met the Valencia criteria for CRPS with remission of some features. No resolution: the patient had no improvement following their primary treatment. All in this category had symptoms of equal or near-equal severity to their original presentation. All of these participants had a secondary treatment prescribed.

All patients were evaluated on this scale to determine the effectiveness of the prescribed course of treatment. Residual symptoms from the CRPS inciting event were included in the categorization of patients. Complete resolution required the resolution of all CRPS symptoms and symptoms relevant to the inciting injury if applicable.

Some residual symptoms were likely a product of the CRPS inciting injury and not directly attributable to the CRPS itself; for example, stiffness with incomplete range of motion in the finger joints. In such instances, patients are not reported as having complete resolution despite the CRPS having resolved because of self-report of ongoing impairment.

### Inclusion Criteria

Participants were included in this cohort study only if they had been clinically diagnosed with CRPS as per the Budapest criteria. Within this cohort, a smaller subset of patients was identified who had undergone electrodiagnosis and had findings indicative of a nerve injury, including peripheral neuropathies, plexopathies, or radiculopathies in the region affected by CRPS, coinciding temporally with their positive CRPS diagnosis.

### Exclusion Criteria

Participants who had positive electrodiagnostic findings only in regions separate from their CRPS were excluded from the study. Participants whose diagnosis was changed from CRPS after further investigation were excluded from the study. Participants who underwent electrodiagnosis but had normal findings were excluded.

## Results

The initial ICD code search yielded 286 charts for review: 248 were diagnosed with CRPS using the Budapest criteria; 60 patients underwent electrodiagnostic studies, and 19 had normal findings. The 41 abnormal studies fit the inclusion criteria (17 males, 24 females). This represents 17% of patients diagnosed with CRPS in the review. The 41 patients were naturally divided into three categories: the first two categories included those where the nerve injury was felt to be contributing or the cause of CRPS and there was either an identifiable traumatic injury or disease onset and those with an atraumatic, more gradual onset, and the third category included a small group of four in which the electrodiagnostic findings were felt to be of delayed onset and not contributing to the CRPS. Nearly all patients included in the study had attended physiotherapy or occupational therapy as part of their normal care.

### Nerve Injury Coincided with CRPS Onset

This first category included 21 participants with electrodiagnostic findings that suggested that the nerve injury coincided with the CRPS inciting event such as an acute trauma that damaged the nerve or disease process. Sixteen of these 21 patients had injuries of the upper limb, 4 the lower limb, and 1 the cervical spine. The median time from the initial trauma to assessment in the clinic was 113 days (σ = 162.05; $\overset{ˉ}{x}$ = 154.67; min = 5; max = 664). Ten participants in this category had fracture as their CRPS inciting event (48%). Other mechanisms included elective surgery, laceration, and infection. Each participant in this category’s presentation is summarized in Table 2.

**Table 2.** Patients whose CRPS onset coincided with the nerve injury’s presentations, treatments, and outcomes sorted by initial treatment, additional treatments, and outcome.

Cause of CRPS	Electrodiagnosis	Initial treatment	Outcome of initial treatment	Additional treatments	Outcome of all additional treatments
Humerus fracture	Brachial plexopathy	Prednisone	Completely resolved	N/A	Completely resolved
Multiple distal arm and hand lacerations, surgical repair	Radial neuropathy, decreased recruitment to extensor pollicis longus	Prednisone	Completely resolved	N/A	Completely resolved
C4–C5 facet joint and transverse process fractures	C5, C6 radiculopathy, denervation to deltoid, biceps	Prednisone	Completely resolved	N/A	Completely resolved
Humerus fracture	Brachial plexopathy, denervation, ulnar neuropathy	Prednisone	Mostly resolved	N/A	Mostly resolved
Disseminated zoster infection	Brachial plexopathy	Prednisone	Mostly resolved	N/A	Mostly resolved
Polytrauma affecting the pelvis, knee, distal leg, ankle, foot, many surgeries	Peroneal neuropathy, plexopathy, denervation to many muscles	Prednisone	Partially resolved	Prednisone	Mostly resolved
Posterior cruciate ligament avulsion	Peroneal neuropathy	Prednisone	Mostly resolved	Prednisone	Mostly resolved
Laceration to flexor tendon and digital nerve	Median neuropathy	Prednisone	Mostly resolved	Alendronate, prednisone	Completely resolved
Clavicle and rib fractures, surgical repair	Brachial plexopathy, denervation, median neuropathy	Prednisone	Mostly resolved	Prednisone, night splint	Mostly resolved
Fall on shoulder, nature of injury unclear	C6 radiculopathy, denervation to deltoid, biceps, extensor digitorum, triceps, pectoralis, supraspinatus	Prednisone	Mostly resolved	Cortisone and lidocaine injections	Mostly resolved
Elective surgery	Median, ulnar neuropathy	Prednisone	Mostly resolved	Surgery, prednisone	Completely resolved
Shoulder dislocation	Brachial plexopathy, denervation	Prednisone	Mostly resolved	Prednisone, surgery	Completely resolved
Distal radius and ulna fracture, surgical repair	Ulnar neuropathy, denervation	Prednisone	Mostly resolved	Surgery	Completely resolved
Distal radius fracture with dislocation, surgical repair	Median and ulnar neuropathy, denervation	Prednisone	Mostly resolved	Surgery	Mostly resolved
Elective surgery	Median and ulnar neuropathy	Prednisone	Partially resolved	Surgery	Mostly resolved
Distal leg fracture, surgical repair	Peroneal neuropathy, Denervation to extensor digitorum brevis	Prednisone	Mostly resolved	Surgery, adapted footwear	Mostly resolved
Elective surgery	Median and ulnar neuropathy	Prednisone	Partially resolved	Surgery, topical lidocaine, diclofenac, and ketamine	Partially resolved
Distal radius fracture	Median neuropathy	Surgery	Not resolved	Cortisone injection, prednisone	Mostly resolved
Degloving injury with significant burns	Median and ulnar neuropathy, denervation	Surgery	Mostly resolved	Prednisone	Mostly resolved
Lisfranc fracture, surgical repair	Peroneal neuropathy, decreased recruitment to extensor digitorum brevis	Adapted foot ware (rejected medication)	Mostly resolved	Trazodone, cyclobenzaprine	Mostly resolved
Humerus fracture, shoulder replacement	Ulnar neuropathy	Occupational therapy	Unknown	N/A	Unknown

Electrodiagnostic findings for category 1 participants included neuropathies, plexopathies, and radiculopathies. The most frequently affected nerves were the median nerve (33%) and ulnar nerve (33%); these nerves were often both affected. Each category 1 patient’s electrodiagnosis is presented in Table 2. Nerve injuries were suspected or confirmed by the referring physician in 12 of 21 charts (57%).

Overall, 19 of the 21 participants had at least a functional recovery after all interventions (90%; 7 completely resolved, 12 mostly resolved). One participant did not return for follow-up and their outcome is unknown. One participant had only partial resolution of their symptoms. Oral prednisone was the initial treatment for 17 of the 21 participants in category 1 (81%). Seven participants improved with corticosteroids alone (oral plus minus corticosteroid injections). All other participants required additional interventions to address their nerve injuries. Surgery was required to resolve nerve injuries in 9 of the 21 participants (43%). Surgery was the initial treatment for just 2 participants; the others had an initial course of oral prednisone first (Table 2).

### CRPS after Chronic Median Entrapment Neuropathy

Category 2 consisted of 16 patients who presented with chronic signs and symptoms of median entrapment neuropathies at the wrist. The median duration of CRPS symptoms before being assessed in the clinic was 92 days (σ = 95.93; $\overset{ˉ}{x}$ = 114.44; min = 39; max = 437). Of the 16 participants, 6 in category 2 had a fracture as the CRPS inciting event (38%) but the CRPS emerged slowly after. Other mechanisms included elective surgery, laceration, carpal tunnel syndrome, and stroke. Two participants had no clear cause for their CRPS. Nerve injuries were reported by the referring physician in 9 of 16 charts (56%).

All participants in this category had at least a functional recovery. Oral prednisone was the initial treatment for all except one patient in this category; this participant had steroid injections instead. Ten participants improved with just steroids (oral or injected), and 4 participants required carpal tunnel decompression surgery to address the nerve injury (Table 3).

**Table 3.** Patients whose nerve injury predated the onset of CRPS’s presentations, treatments, and outcomes sorted by initial treatment, additional treatments, and outcome.

Cause of CRPS	Electrodiagnosis	Initial treatment	Outcome of initial treatment	Additional treatments	Outcome of all additional treatments
Distal radius fracture	Median neuropathy	Prednisone	Completely resolved	N/A	Completely resolved
Laceration, surgical repair	Median neuropathy	Prednisone	Completely resolved	N/A	Completely resolved
Elective surgery	Median neuropathy	Prednisone	Completely resolved	N/A	Completely resolved
No clear cause	Median neuropathy	Prednisone	Mostly resolved	N/A	Mostly resolved
Distal radius fracture	Median neuropathy	Prednisone	Mostly resolved	N/A	Mostly resolved
Distal radius fracture	Median neuropathy	Prednisone	Mostly resolved	N/A	Mostly resolved
No clear cause	Median neuropathy	Prednisone	Partially resolved	Prednisone	Completely resolved
Elective surgery	Median neuropathy	Prednisone	Mostly resolved	Prednisone, cortisone injection	Completely resolved
Distal radius fracture	Median neuropathy	Prednisone	Mostly resolved	Cortisone injection	Mostly resolved
Stroke	Median neuropathy	Prednisone	Partially resolved	Cortisone injections, prednisone	Completely resolved
No clear cause	Median neuropathy	Prednisone	Mostly resolved	Referred to rheumatologist	Mostly resolved
Carpal tunnel syndrome release surgery	Median neuropathy	Prednisone	Mostly resolved	Topical diclofenac, lidocaine, clonidine	Mostly resolved
Distal radius fracture	Median neuropathy	Prednisone	Mostly resolved	Surgery	Completely resolved
Distal radius fracture	Median neuropathy	Prednisone	Mostly resolved	Prednisone, surgery	Mostly resolved
Elective surgery	Median neuropathy	Prednisone	Partially resolved	Topical lidocaine, ketamine, clonidine, surgery	Mostly resolved
Carpal tunnel syndrome	Median neuropathy	Cortisone injection	Mostly resolved	Surgery	Mostly resolved

### Nerve Injury Emerged after CRPS Onset

The final category of four participants had late onset of nerve symptoms, long after their CRPS symptoms emerged; thus, the nerve injury was not felt to be a cause of the CRPS. All four had injuries affecting the upper limb. The median duration of CRPS symptoms before being assessed in the clinic was 71.50 days (σ = 20.80; $\overset{ˉ}{x}$ = 78; min = 61; max = 108). CRPS was caused by fracture in two cases, a dislocation in one case, and one case had no clear cause. No nerve injuries were suspected by any referring physicians; electrodiagnosis revealed median or ulnar neuropathies in all participants in this category.

All participants in this category had at least a functional recovery after all treatments. All participants in this category had oral prednisone as the initial treatment. All but one participant in this category responded to oral prednisone alone. The final participant required surgery to address their nerve injury Table 4.

**Table 4.** Patients whose CRPS onset predated nerve injury’s presentations, treatments, and outcomes sorted by initial treatment, additional treatments, and outcome.

Cause of CRPS	Electrodiagnosis	Initial treatment	Outcome of initial treatment	Additional treatments	Outcome of all additional treatments
Glenohumeral dislocations	Ulnar neuropathy, denervation	Prednisone	Completely resolved	N/A	Completely resolved
No clear cause	Median neuropathy	Prednisone	Mostly resolved	N/A	Completely resolved
Distal radius fracture, surgical repair	Median neuropathy	Prednisone	Mostly resolved	Prednisone	Mostly resolved
Wrist fracture	Median neuropathy	Prednisone	Partially resolved	Topical diclofenac, physiotherapy, prednisone, surgery	Mostly resolved

## Discussion

This retrospective review analyzed the 41 patients who were diagnosed with discrete nerve injuries using electrodiagnosis (17%), out of 248 patients with CRPS. However, only 51% of the 41 referrals referenced a suspected nerve injury. Nearly all patients had a course of oral prednisone or corticosteroid injection; however, more than 30% of all patients required surgical interventions to resolve their discrete nerve injuries. Following our algorithm of oral prednisone, cortisone injections, and surgery, 24% participants had complete resolution of CRPS symptoms, and an additional 71% had a near-complete resolution of CRPS symptoms. Overall, 95% of the cohort reported at least a functional recovery from CRPS. It is important to note that in patients whose inciting injury was traumatic, residual effects from this traumatic injury or joint contractures are factors that could have prevented a full recovery.

Our patient population is in alignment with the expected epidemiology of CRPS.^1–3^ A Mayo Clinic study found that fracture is the leading cause of CRPS, accounting for 46% of all cases.^11^ CRPS affects females more than males, and upper extremities are more commonly affected.^3,11,12^ In our cohort, 46% of patients had a fracture preceding CRPS, 59% were women, and 88% had upper extremity involvement.

A discussion of the evidence for using corticosteroids is not the objective of this article, because oral prednisone has been noted to be effective in managing acute CRPS symptoms.^8,13–15^ Because most patients in our cohort were seen within 6 months of onset of CRPS, lumbar and cervical sympathetic blocks were not utilized because blocking the sympathetic nervous system is no longer considered a first-line therapy.^5,14,15^ In our cohort of patients with discrete nerve injuries, many patients did not respond fully to corticosteroids alone. Surgery for discrete nerve injuries was required for 34% of the 41 participants.

Without a neurological assessment, a nerve injury may not be identified. The inflammation of CRPS may hide the tell-tale signs of the injury and be revealed only after the treatment of swollen inflammatory features. For example, first dorsal interossei (FDI) wasting, indicative of ulnar neuropathy, is camouflaged by edema in Figure 2. The lack of recognition of the presence of CRPS type II could negate the opportunity for a nerve-specific treatment, such as a surgical decompression at Guyon’s canal for this example. In some patients whose nerve injury predated CRPS onset, surgery may have been an additional inciting trauma; CRPS II has been linked to carpal tunnel release surgeries.^16–18^ In this group, treatment with oral prednisone led to positive outcomes, likely due to its documented benefits for neuritis and similar compression conditions.^19,20^ However, four of these patients still required a surgical release for functional recovery (25%). In patients whose nerve injury emerged after the onset of CRPS, we hypothesize that entrapment neuropathy may have been a mechanical sequela of the swelling induced by CRPS I and not the cause of the CRPS. One participant in this group required a surgical intervention (25%). Surgical intervention or corticosteroid injections should be considered in the treatment of CRPS II, especially when an optimal outcome is not achieved from an initial course of oral prednisone.

Electrodiagnostic testing in patients with CRPS revealed that the distinction between CRPS I and II is not straightforward. The 14 patients who required surgical decompression demonstrate the precise relationship between ongoing CRPS symptoms and discrete nerve injury: both must be resolved for symptoms to abate. It is notable that the anti-inflammatory principles of prednisone can influence nerve injury caused by compression, as evidenced by the use of corticosteroids in the treatment of carpal tunnel syndrome.^21,22^ For disease processes such as neuritis, corticosteroids are considered to be first-line treatment in the acute phase.^19,20^

There are limitations to this study. All patients were treated by the same physical medicine and rehabilitation specialist, and almost all of the nerve studies were performed by them. Not all patients can tolerate electrodiagnosis, particularly prior to treatments, and swelling may make studies technically difficult. This study followed a cohort of only 41 participants, and data were compiled retrospectively, though the sample size included in this study is comparable to that of many other published CRPS studies, which remains a poorly understood and underinvestigated condition.

## Conclusions

CRPS remains a challenging diagnosis due to the condition’s progressive nature. Electrodiagnosis could improve diagnosis and treatment outcomes for patients with CRPS. In this study, 60 of 248 patients diagnosed with CRPS underwent electrodiagnostic findings, 41 of whom had positive findings. Only 51% of the nerve injuries were identified by referring physicians. The presentation of CRPS can hide the tell-tale signs of nerve injury, and nerve-specific interventions like surgical decompression or targeted cortisone injections may be required for complete resolution. Therefore, electrodiagnosis should be considered for patients with CRPS in the acute or subacute phase who do not respond to initial treatment with corticosteroids and or rehabilitation.
